# Research on Herbal Therapies for Osteoarthritis in 2004–2022: A Web of Science-Based Cross-Sectional Bibliometric Analysis

**DOI:** 10.1155/2022/6522690

**Published:** 2022-07-30

**Authors:** Weijiang Song, Jiaqi Chen, Guoyan Yang, Jiahe Liao, Hongbo Shen, Sai Li, Ning Ding, Dong Li

**Affiliations:** ^1^Traditional Chinese Medicine Department, Peking University Third Hospital, Beijing, China; ^2^Graduate School, Beijing University of Chinese Medicine, Beijing, China; ^3^Traditional Chinese Medicine Department of Rheumatism, China-Japan Friendship Hospital, Beijing, China; ^4^NICM Health Research Institute, Western Sydney University, Sydney, Australia

## Abstract

**Objective:**

The extent, range, and nature of available research in the field of herbal therapies for osteoarthritis (OA) have not been systematically analyzed. This study aimed to map the literature available on herbal therapies for OA and identify global hotspots and trends in this field.

**Methods:**

Studies on herbal therapies for OA published between 2004 and 2022 were searched from the Web of Science Core Collection. Microsoft Excel, SPSS Statistics, and CiteSpace software were used to analyze and visualize the quantity and citations of publications, and the research hotspots and trends in research on herbal therapies for OA.

**Results:**

A total of 1649 publications mainly from 76 countries/regions and 270 institutions were included in this study. From 2004 to 2022, there is an upward trend in the publications of herbal therapies for OA. China ranked first in the number of publications (*n* = 568, 34.45%), followed by the USA (*n* = 353, 21.41%), South Korea (*n* = 187, 11.34%), Germany (*n* = 85, 5.15%), and England (*n* = 79, 4.79%). Kyung Hee University (*n* = 46), Xianxiang Liu (*n* = 25), and *Evidence-Based Complementary and Alternative Medicine* (*n* = 74) were the most prolific affiliation, author, and journal, respectively. Felson DT (*n* = 185) and *Arthritis and Rheumatism* (*n* = 1173) held the record for the most cited papers by an author and journal, respectively. Currently, the hot keywords in the field of herbal therapies for OA include knee OA, traditional Chinese medicine (TCM), differentiation, rosa canina, inflammation, oxidative stress, stem cell, and regenerative medicine. The emerging research trends in herbal therapies for OA are herbal medicinal product, chronic knee pain, mesenchymal stem cell, and clinical pharmacology.

**Conclusions:**

Research on herbal therapies for OA is flourishing, but communication among countries/regions should be strengthened. Current research on herbal therapies for OA mainly focuses on knee OA, TCM, differentiation, rosa canina, inflammation, oxidative stress, stem cell, and regenerative medicine. The research frontiers are herbal medicinal product, chronic knee pain, mesenchymal stem cell, and clinical pharmacology.

## 1. Introduction

Osteoarthritis (OA) is the most common degenerative joint disorder that affects one or more diarthrodial joints and is often accompanied by joint pain, stiffness, dysfunction, and structural damages [1]. Age, obesity, genetics, sex, and joint biomechanics are generally considered risk factors for OA [2]. With the ageing and increasing obesity in the global population and the increasing numbers of joint injuries, OA is prevalent, with a worldwide estimate suggesting that 250 million people are currently affected [3]. Moreover, according to the *Global Burden of Disease Study 2019*, OA is the leading cause of disability and source of societal cost in older adults [4].

Recent research has revealed that the mechanism of OA is closely related to inflammation, oxidative stress, apoptosis, and energy metabolism, and these factors can interact with each other [5]. However, the pathogenesis of OA has not been understood fully, and further research is still needed. To date, no disease-modifying OA drug has received regulatory approval yet. For the main symptoms of joint pain, nonsteroidal anti-inflammatory drugs (NSAIDs), analgesics, and corticosteroids are available as optional drugs. Even so, the cardiovascular and digestive safety issues often surround NSAIDs, and the addictive nature of central analgesics are also concerns of clinicians and patients [6, 7]. Recent case series suggested that negative structural outcomes including accelerated OA progression, subchondral insufficiency fracture, complications of osteonecrosis, and rapid joint destruction may be observed in patients receiving intra-articular corticosteroid injections [8]. Therefore, there is a need to explore effective and safer treatments. Herbs or herbal products have a long history in treating OA and show great potential to generate less adverse events than pharmaceutical drugs [9, 10]. Furthermore, researchers have discovered that herbal therapies can slow down the progression of OA via several mechanisms [11]. At present, there are a large number of literature on herbal therapies for OA, and a bibliometrics is needed.

Bibliometrics is a cross-science that uses mathematical and statistical methods to study documents and bibliometric characteristics [12, 13], such as countries/regions, institutions, journals, authors, and citations. [14]. So far, there are few bibliometric studies on traditional medicine in treating OA, and the majority of them focused on nondrug treatments, such as Tai Chi and acupuncture [15, 16]. However, no bibliometric study has been published on herbal therapies for OA to inform the volume, breadth, and characteristics of research in this area. Hence, we aimed to analyze publications on herbal therapies for OA from 2004 to 2022 and discussed the current hotspots and trends in research.

## 2. Materials and Methods

### 2.1. Data Collection

We searched the Web of Science Core Collection (WoSCC) from its inception until 10 February 2022. The search terms were centered on OA and herbs (Figure S1). Articles and reviews reporting herbal therapies in treating OA were eligible for inclusion irrespective of language. Early access, conference proceedings, letters, editorial materials, corrections, book chapters, retracted publications, and editorial materials were excluded. Two authors independently screened all titles and abstracts of the records. The full texts were retrieved for further identification in accordance with eligibility criteria. All uncertainties or discrepancies were resolved through discussion. A total of 1,743 records were identified, of which 94 records were excluded because they did not meet the eligibility criteria. Finally, 1,649 papers were included in this bibliometric study (Figure S1).

### 2.2. Data Analysis

All included papers retrieved from WoSCC were imported into Microsoft Excel 2019 (Microsoft Corp, Redmond, Washington, USA), which was used to analyze the number of papers published in the year and journal. SPSS Statistics 20 (IBM, Armonk, New York, USA) was used for trend analysis of the number of publications. A *p* < 0.05 was considered statistically significant. CiteSpace V5.8.R3 (Chaomei Chen, Philadelphia, Pennsylvania, USA) was used to perform visual analysis, including the distribution of countries/regions, institutions, authors, co-cited authors, cocited journals, cocited references, keyword cluster, and timeline viewer. CiteSpace is a widely used visualization analysis software, which can combine information visualization methods, bibliometrics, and data mining algorithms in an interactive visualization tool for extraction of patterns in citation data [17, 18] to analyze the structure and distribution of scientific knowledge and the visualizing trends in scientific literature [19].

## 3. Results

### 3.1. The Trend of Publication Outputs

A total of 1,649 papers involving 1,285 articles and 364 reviews published between January 2004 and February 2022 were included for analysis in this study. From 2004 to 2022, the total number of published papers on herbal therapies for OA increased year by year (Figure S2, *p* < 0.001). Since 2017, the number of outputs in the field of herbal therapies for OA has exceeded 100, and the number of publications increased significantly in 2020, with a total number of 235 (14.3%). Compared with 2020, the number of publications in 2021 had a slight decrease. However, till February 10, 2022, 19 papers on herbal therapies for OA have been published in 2022, which was similar to a year of outputs in 2006.

### 3.2. Distribution of Countries/Regions and Institutions

The 1,649 included papers were published mainly by 76 countries/regions involving 270 institutions. As shown in Table 1, China ranked first in the number of publications (*n* = 568, 34.45%), much higher than other countries/regions, followed by the USA (*n* = 353, 21.41%), South Korea (*n* = 187, 11.34%), Germany (*n* = 85, 5.15%), and England (*n* = 79, 4.79%). Kyung Hee University was the most prolific institution (*n* = 46, 2.79%), followed by Shanghai University of Traditional Chinese Medicine (*n* = 42, 2.55%), Fujian University of Traditional Chinese Medicine (*n* = 39, 2.37%), Beijing University of Chinese Medicine (*n* = 32, 1.94%), and Zhejiang University (*n* = 27, 1.64%).

**Figure 1:**
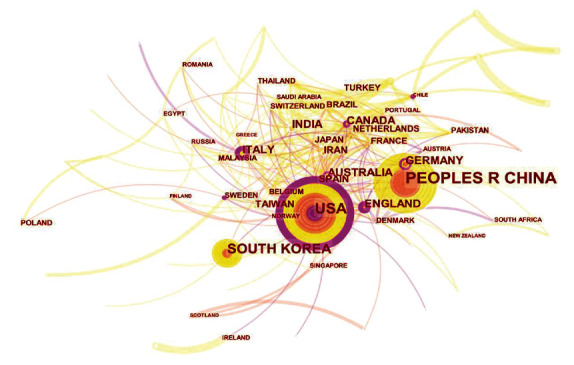
CiteSpace visualization map of publications in the field of herbal therapies for OA from different countries/regions.

Figure 1 showed the visualization map of publications related to herbal therapies for OA from different countries/regions. Each node represents a country. The lines between two nodes suggest the communication capability between two countries, and the denser lines indicate closer communication. Overall, the centrality of nodes in the knowledge map measures the importance of node position and reflects the communication capability of the nodes, and each node with high centrality has an outer purple circle. Our results showed that among the top 10 countries with the highest number of publications, 6 countries (USA, Italy, England, Canada, Australia, and Germany) had a centrality of more than 0.1, suggesting these are important bridge countries having strong communication power all over the world in the field of herbal therapies for OA.

### 3.3. Authors and Co-Cited Authors

There were 2,038 major authors involved in the 1,649 papers in this study. As shown in Table 2, Xianxiang Liu was the most prolific author in the number of publications on herbal therapies for OA (*n* = 25, 1.52%), followed by Hongzhi Ye (*n* = 22, 1.33%), and Xihai Li (*n* = 20, 1.21%). Cocited authors are two or more authors who are cited by another literature at the same time. There were 1,848 cocited authors in this study. Among these cocited authors, 9 had a frequency of citation over 100 times, and Felson DT was the top cocited author (*n* = 185), followed by Zhang W (*n* = 157).

### 3.4. Journals and Cited Journals

A total of 586 journals were involved in the 1,649 articles in this study. As shown in Table 3, *Evidence-Based Complementary and Alternative Medicine* was the top journal in the number of publications on herbal therapies for OA (*n* = 74, 4.49%), followed by the *Journal of Ethnopharmacology* (*n* = 56, 3.40%) and *Medicine* (*n* = 49, 2.97%). Cocited journals are two or more journals that are cited simultaneously. Among 924 cocited journals, 5 were cited over 500 times. *Arthritis and Rheumatism* was the top cocited journal, followed by *Osteoarthritis and Cartilage* (*n* = 888), and *Annals of The Rheumatic Diseases* (*n* = 667).

### 3.5. The Hotspots of Keywords

Keywords often reflect the research topics, and their analysis can indicate the research hotspots in a specific field. The keywords (*n* ≥ 80) related to herbal therapies for OA are shown in Table S1. Among these keywords, OA had the highest frequency (*n* = 447), followed by knee OA (*n* = 438), double-blind (*n* = 202), rheumatoid arthritis (*n* = 187), expression (*n* = 170), and pain (*n* = 159). OA, knee OA, and double-blind had a high centrality (>0.10), indicating a great influence of these keywords in the research of herbal therapies for OA.

**Figure 2:**
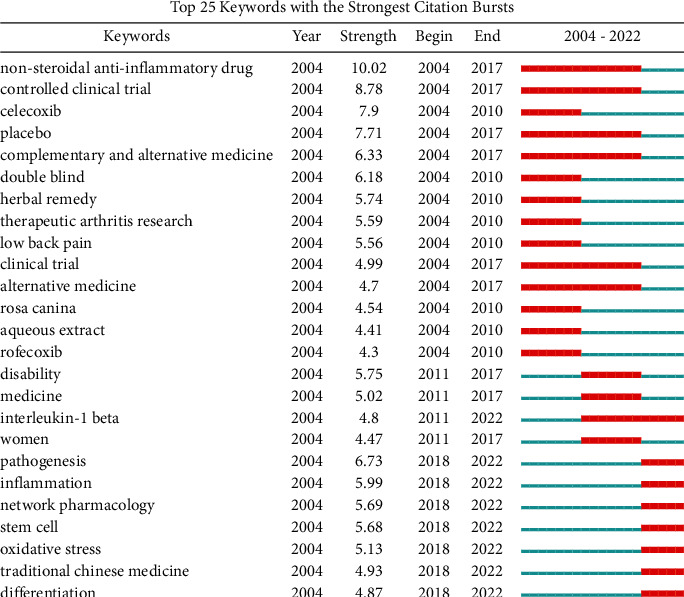
CiteSpace visualization map of top 25 keywords with the strongest citation bursts involved in herbal therapies for OA.

The strong citation burst of keywords represents sharp changes in the number of citations for keywords, which can reflect the rise or fall of research hotspots. As shown in Figure 2, among the top 25 keywords related to herbal therapies for OA, NSAIDS, controlled clinical trial, placebo, and complementary and alternative medicine were popular keywords from 2004 to 2017. Since 2011, interleukin-1*β* has been a hot keyword. Since 2018, pathogenesis, inflammation, network pharmacology, stem cell, oxidative stress, traditional Chinese medicine (TCM), and differentiation have been hotspots studied in herbal therapies for OA.

**Figure 3:**
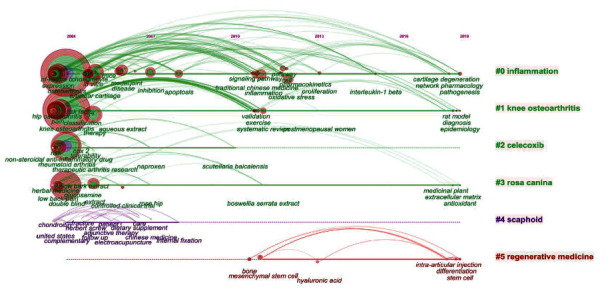
CiteSpace visualization map of timeline viewer related to herbal therapies for OA.

The timeline mapping of keywords is the summarization and classification of research keywords, which can show the characteristics of research over time. As shown in Figure 3, keywords on herbal therapies for OA were classified into the following 6 clusters: inflammation, knee OA, celecoxib, rosa canina, scaphoid, and regenerative medicine. Since 2004, inflammation, knee OA, and rosa canina have been in the spotlight. Celecoxib and scaphoid boomed in 2004 but declined in 2010 and 2007, respectively. Regenerative medicine began to garner attention in 2010 and has continued to develop in recent years.

### 3.6. The Research Frontiers of Herbal Therapies for OA

**Figure 4:**
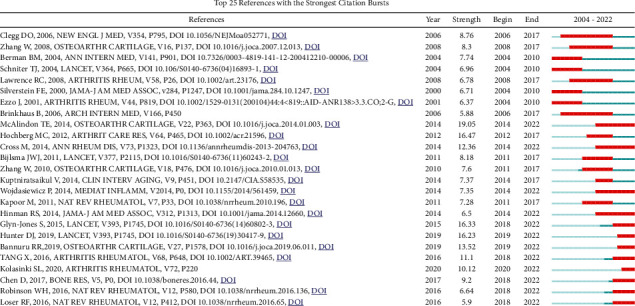
CiteSpace visualization map of top 25 references with the strongest citation bursts involved in herbal therapies for OA.

Cocited papers mean two papers appear in the reference list of another paper, and cocitation analysis can mine the cocitation relationships of papers in the repository. A total of 1,373 articles were cited in papers on herbal therapies for OA in this study, of which 10 papers were cited more than 20 times (table S2). The paper ranked at the top of citations was *OARSI guidelines for the non-surgical management of knee osteoarthritis*. The strongest citation bursts of references, just as the strongest citation bursts of keywords, can reflect changes in research hotspots over a period of time.  Figure 4 showed the top 25 references with the strongest citation bursts involved in herbal therapies for OA. Up to now, nearly half of the references remain research hotspots.

On the basis of cocitation analysis, we used CiteSpace to extract cluster labels from papers by cluster analysis, showing the research frontiers in the field of herbal therapies for OA. Silhouette score can evaluate the effect of clustering. When the silhouette score is >0.7, the clustering result is convincing. As shown in Table S3, the following 7 main cluster labels were obtained in the field of herbal therapies for OA: network pharmacology, chronic disorder, mesenchymal stem cell, clinical pharmacology, herbal medicinal product, biological basis, and chronic knee pain.

## 4. Discussion

### 4.1. General Information

This is the first bibliometric analysis identifying the global research hotspots and trends in the field of herbal therapies for OA. Our results indicated that the number of publications on herbal therapies for OA presented an overall upward trend in the field of herbal therapies for OA from 2004 to 2022, suggesting that research on herbal therapies for OA is flourishing. Between 2004 and 2016, the number of publications grew slowly. Since 2016, the growth had accelerated, especially in 2020, suggesting that herbal therapies for OA is attracting increasing attention in recent years.

We found that China ranked first in the number of publications on herbal therapies for OA, followed by the USA, South Korea, Germany, and England. Meanwhile, the centrality of the USA was significantly higher than other countries, indicating that the USA played a bridging role in international cooperation. Seven of the top 10 institutions in the number of publications were from China, with Shanghai University of Traditional Chinese Medicine and the Chinese University of Hong Kong showing a high level of collaboration. Two Korean institutions, named Kyung Hee University and Korea Institute of Oriental Medicine, were on the top 10 lists, with less international collaboration. Xianxiang Liu and *Evidence-Based Complementary and Alternative Medicine* were the most prolific author and journal, respectively. Simultaneously, Felson DT and *Arthritis and Rheumatism* were the most influential author and journal by holding the most cited papers, respectively. There was no overlap in the top 10 publications and citations by authors and journals, revealing that we still lack author and journal with a high impact on herbal therapies for OA. The *OARSI guidelines for the non-surgical management of knee osteoarthritis* [20] published in 2019 was the most cited reference, while a new guideline published in 2020 named *the 2019 American College of Rheumatology/Arthritis Foundation Guideline for the Management of Osteoarthritis of the Hand, Hip, and Knee* may be a new focus in the future [21].

### 4.2. The Hotspots and Frontiers

#### 4.2.1. Role of Herbal Therapies for OA

In research on herbal therapies for OA, our results indicated that knee OA has been a research hotspot for a long time (since 2004), and TCM and differentiation also became a research hotspot in 2018. All these suggested that traditional Chinese herbal medicine are research hotspots in the field of herbal therapies for OA. In TCM theories, differentiation refers to syndrome identification, which means the classification of syndrome into a variety of unbalance conditions [22]. Patients with OA could have distinct syndrome classification that dictates different treatment strategies. In this case, traditional Chinese herbal medications will be tailored to the individual patient, which may result in better effects and fewer side effects. However, due to the individualized characteristics of the syndrome, it is difficult to conduct randomized controlled trials and placebo-controlled trials for traditional Chinese herbal medicine. This may account for the decline in randomized placebo-controlled trials since 2018. Due to a lack of high-quality clinical evidence, Chinese herbal medications are often considered complementary or alternative therapies for patients with OA.

Herbal medicinal products and chronic knee pain are research frontiers in herbal therapies for OA. As one of the major symptoms of OA, chronic knee pain seriously impairs the quality of life for patients [23]. Many studies have indicated that herbal therapies can improve pain and slow the progression of OA [24]. Some researchers aim at transforming herbs with potential therapeutic effects into established herbal medicinal products or new clinical medicines. However, the selection of herbs for the treatment of OA is largely empirical, is time-consuming, and lacks high-quality evidence to support it. The complex composition of herbs makes it difficult to explore the exact active ingredient. Furthermore, the interactions between herbal medications with each other are not fully understood. Therefore, there is a long way to elucidate the mechanisms and determine the efficacy and role of herbal therapies for OA.

#### 4.2.2. Main Types of Herbal Therapies for OA

There is a wide range of herbs available for the treatment of OA. Our results showed that rosa canina, a European traditional herb, has been extensively studied since 2004 [25]. A recent meta-analysis included randomized controlled trials that showed that rosa canina powder can alleviate the pain of OA [26], and its mechanism of action is related to antioxidant and anti-inflammatory [27]. TCM mainly includes herbal compounds and herb extracts and has been used in China for over 2,500 years for chronic pain and OA. A famous TCM compound named *Duhuo Jisheng Tang*, which was invented in the Tang Dynasty, is considered beneficial and widely used in treating OA in China. In addition to compound herbal medicine, many single herbs also showed effects on attenuating symptoms of OA and may be potential therapeutic agents for OA, such as *rhizoma drynariae*, *icariin*, and *curcuma longa* [28–30]. However, current herbal medicines are primarily used as complementary or alternative therapies for OA due to limited evidence [31]. Further long-term, large-sample, high-quality evidence is warranted to support the application of herbal medicines for the treatment of OA.

#### 4.2.3. Mechanisms of Herbal Therapies for OA

For the mechanisms of herbal therapies in treating OA, our results showed that inflammation, oxidative stress, stem cell, regenerative medicine, and network pharmacology were important research hotspots; mesenchymal stem cell and clinical pharmacology were major research frontiers. Understanding the mechanisms of herbal therapies is helpful to maximize the therapeutic effects and develop therapeutic agents. Network pharmacology and clinical pharmacology are research methods aiming at exploring pharmacological actions and therapeutic mechanisms of herbal medicines for OA. It is worth noting that network pharmacology must be analyzed in conjunction with experimental validation to generate trusted evidence.

Inflammation, interleukin-1*β*, and oxidative stress have been research hotspots in the mechanism study of herbal therapies for OA for a long time. As a proinflammatory factor, interleukin-1*β* can induce joint inflammation and seems to be associated with cartilage destruction [32]. Interleukin-1*β* is now often used as a stimulator in cellular experiments. Oxidative stress has been proposed as a driver of the catabolic and anabolic signaling imbalance in cartilage that results in progressive matrix degradation [33]. In addition, oxidative stress can induce senescence in joint cells [34]. There was a correlation between increased oxidative stress and the induction of senescence in cartilage, which might drive OA [33]. Some herbs, such as *curcuma longa*, *vernonia amygdalina*, and *icariin*, can suppress inflammation, decrease oxidative stress, and reduce local symptoms of joints in OA [29, 35, 36].

Stem cell was a research hotpot, and mesenchymal stem cell and regenerative medicine were research frontiers in herbal therapies for OA. Regenerative medicine is an emerging research field in recent years. Recent studies suggested that bone mesenchymal stem cell therapy can relieve local pain and partially repair injured cartilage, with the therapeutic goal of joint regeneration [37]. Some herbs (such as *andrographolide* and *honokiol*) have been shown to improve cell survival and chondrogenesis of mesenchymal stem cells [38, 39], which may be a major potential therapeutic mechanism of herbal therapies for OA.

### 4.3. Limitations

There are certain shortcomings in this study. Firstly, since we used CiteSpace software for data analysis, which removed some components that were calculated to be insignificant during the analyses, our results may miss some interesting data. Secondly, we only searched one comprehensive English database that could not cover all studies in herbal therapies for OA, so potential bias may exist in the results. Thirdly, as this is a bibliometric analysis, we did not evaluate the methodological quality of included studies and critically analyze the effects of herbal therapies for OA; our results were based majorly on the quantity of publications and citations and may use as a start point for research in this area.

## 5. Conclusion

Research on herbal therapies for OA is flourishing, but communication among countries should be strengthened. China ranked first in the number of publications, followed by the USA, South Korea, Germany, and England. Current research on herbal therapies for OA mainly focuses on knee OA, TCM, differentiation, rosa canina, inflammation, oxidative stress, stem cell, and regenerative medicine. The emerging research trends in herbal therapies for OA are herbal medicinal product, chronic knee pain, mesenchymal stem cell, and clinical pharmacology.

## Figures and Tables

**Table 1:** Distribution of publications in the field of herbal therapies for OA from different countries/regions and institutions.

Rank	Country/region	Year	Centrality	Count (%)	Institution	Year	Centrality	Count (%)
1	Peoples R China	2004	0.04	568 (34.45)	Kyung Hee Univ	2005	0.06	46 (2.79)
2	USA	2004	0.51	353 (21.41)	Shanghai Univ Tradit Chinese med	2011	0.14	42 (2.55)
3	South Korea	2004	0.05	187 (11.34)	Fujian Univ Tradit Chinese med	2010	0.02	39 (2.37)
4	Germany	2004	0.12	85 (5.15)	Beijing Univ Chinese med	2015	0.03	32 (1.94)
5	England	2004	0.23	79 (4.79)	Zhejiang Univ	2012	0.06	27 (1.64)
6	India	2004	0.08	71 (4.31)	Shanghai Jiao Tong Univ	2011	0.01	25 (1.52)
7	Australia	2004	0.19	67 (4.06)	Peking Univ	2009	0.07	24 (1.46)
8	Italy	2004	0.31	67 (4.06)	Chinese Univ Hong Kong	2004	0.15	24 (1.46)
9	Canada	2004	0.15	57 (3.46)	Univ Sydney	2011	0.08	23 (1.39)
10	Taiwan	2007	0.05	47 (2.85)	Korea Inst Oriental med	2006	0.05	23 (1.39)

**Table 2:** Top authors (*n* ≥ 10) and co-cited authors (*n* ≥ 80) related to herbal therapies for OA.

Rank	Author	Year	Centrality	Count (%)	Cocited author	Year	Centrality	Citation
1	Xianxiang Liu	2009	<0.01	25 (1.52)	Felson DT	2004	<0.01	185
2	Hongzhi Ye	2009	<0.01	22 (1.33)	Zhang W	2004	0.04	157
3	Xihai Li	2011	0.01	20 (1.21)	Anonymous	2005	<0.01	153
4	Chunsong Zheng	2011	0.01	15 (0.91)	Hochberg MC	2004	0.03	151
5	Dongsuk Park	2008	<0.01	11 (0.67)	Bellamy N	2004	0.01	145
6	Yonghyeon Baek	2008	<0.01	11 (0.67)	Goldring MB	2005	<0.01	143
7	Peijian Tong	2014	<0.01	11 (0.67)	Altman R	2004	0.01	142
8	Jaedong Lee	2008	<0.01	10 (0.61)	Altman RD	2004	<0.01	125
9	Guangwen Wu	2011	0.01	10 (0.61)	Mcalindon TE	2004	0.01	121
10	Xueyong Shen	2013	<0.01	10 (0.61)	Loeser RF	2008	0.01	98
11	Qi Jia	2010	<0.01	10 (0.61)	Ernst E	2004	<0.01	95
12	Ling Zhao	2013	<0.01	10 (0.61)	Hunter DJ	2008	<0.01	88
13	Huifeng Xu	2011	<0.01	10 (0.61)	Lawrence RC	2004	<0.01	82

**Table 3:** Top 10 journal and cocited journals related to herbal therapies for OA.

Rank	Journal	Count (%)	Cocited journal	Citation
1	Evidence-Based Complementary and Alternative Medicine	74 (4.49)	Arthritis and Rheumatism	1,173
2	Journal of Ethnopharmacology	56 (3.40)	Osteoarthritis and Cartilage	888
3	Medicine	49 (2.97)	Annals of the Rheumatic Diseases	667
4	Phytotherapy Research	29 (1.76)	Journal of Rheumatology	555
5	BMC Complementary and Alternative Medicine	24 (1.46)	Lancet	502
6	Trials	23 (1.39)	Arthritis Research & Therapy	474
7	Frontiers in Pharmacology	21 (1.27)	Rheumatology	431
8	Chinese Journal of Integrative Medicine	20 (1.21)	PloS One	429
9	Osteoarthritis and Cartilage	19 (1.15)	Journal of Ethnopharmacology	421
10	Phytomedicine	19 (1.15)	Annals of Internal Medicine	379

## Data Availability

Data sharing is not applicable to this article as no new data were created in this study. These datasets were derived from the following public domain resources: https://www.webofscience.com/wos/woscc/advanced-search.

## Supplementary Materials

Supplementary Materials Figure S1 Flowchart of literature search and selection. Figure S2 Trends of publications in the field of herbal therapies for OA from 2004 to 2022. Table S1 Top keywords (*n* ≥ 80) related to herbal therapies for OA. Table S2 Top 10 references related to herbal therapies for OA. Table S3 The clusters of cocited references in herbal therapies for OA.
