# Supplementary material for: Research on Herbal Therapies for Osteoarthritis in 2004–2022: A Web of Science-Based Cross-Sectional Bibliometric Analysis
Source: Evid Based Complement Alternat Med. 2022 Jul 30;2022:6522690. doi: 10.1155/2022/6522690 (PMC9356781; doi:10.1155/2022/6522690)
Supplement: Supplementary Materials — Figure S1 Flowchart of literature search and selection. Figure S2 Trends of publications in the field of herbal therapies for OA from 2004 to 2022. Table S1 Top keywords (n ≥ 80) related to herbal therapies for OA. Table S2 Top 10 references related to herbal therapies for OA. Table S3 The clusters of cocited references in herbal therapies for OA. [file 6522690.f1.zip › Table S1 (1).docx]

TABLE S1 Top Keywords (n≥80) related to herbal therapies for OA.

| **Rank** | **Keywords** | **Year** | **Centrality** | **Count** | **Rank** | **Keywords** | **Year** | **Centrality** | **Count** |
| --- | --- | --- | --- | --- | --- | --- | --- | --- | --- |
| 1 | osteoarthritis | 2004 | 0.12 | 447 | 10 | NF-kappa b | 2004 | 0.04 | 125 |
| 2 | knee osteoarthritis | 2004 | 0.14 | 438 | 11 | articular cartilage | 2005 | 0.09 | 123 |
| 3 | double blind | 2004 | 0.17 | 202 | 12 | randomized controlled trial | 2004 | 0.04 | 114 |
| 4 | rheumatoid arthritis | 2004 | 0.08 | 187 | 13 | arthritis | 2004 | 0.06 | 111 |
| 5 | expression | 2004 | 0.09 | 170 | 14 | in vitro | 2005 | 0.08 | 109 |
| 6 | pain | 2004 | 0.06 | 159 | 15 | therapy | 2005 | 0.06 | 109 |
| 7 | hip osteoarthritis | 2004 | 0.06 | 143 | 16 | cartilage | 2004 | 0.04 | 105 |
| 8 | efficacy | 2004 | 0.07 | 130 | 17 | non-steroidal anti-inflammatory drug | 2004 | 0.06 | 84 |
| 9 | management | 2004 | 0.04 | 130 | 18 | inflammation | 2011 | 0.01 | 83 |
