# Supplementary material for: Research on Herbal Therapies for Osteoarthritis in 2004–2022: A Web of Science-Based Cross-Sectional Bibliometric Analysis
Source: Evid Based Complement Alternat Med. 2022 Jul 30;2022:6522690. doi: 10.1155/2022/6522690 (PMC9356781; doi:10.1155/2022/6522690)
Supplement: Supplementary Materials — Figure S1 Flowchart of literature search and selection. Figure S2 Trends of publications in the field of herbal therapies for OA from 2004 to 2022. Table S1 Top keywords (n ≥ 80) related to herbal therapies for OA. Table S2 Top 10 references related to herbal therapies for OA. Table S3 The clusters of cocited references in herbal therapies for OA. [file 6522690.f1.zip › Table S2 (1).docx]

TABLE S2 Top 10 references related to herbal therapies for OA.

| **Rank** | **Title** | **DOI** | **Year** | **Centrality** | **Citation** |
| --- | --- | --- | --- | --- | --- |
| 1 | OARSI guidelines for the non-surgical management of knee osteoarthritis | 10.1016/j.joca.2014.01.003 | 2014 | 0 | 48 |
| 2 | Osteoarthritis | 10.1016/S0140-6736(14)60802-3 | 2015 | 0.03 | 41 |
| 3 | American College of Rheumatology 2012 recommendations for the use of nonpharmacologic and pharmacologic therapies in osteoarthritis of the hand, hip, and knee | 10.1002/acr.21596 | 2012 | 0 | 39 |
| 4 | Osteoarthritis | 10.1016/S0140-6736(19)30417-9 | 2019 | 0.01 | 35 |
| 5 | The Prevalence of Symptomatic Knee Osteoarthritis in China: Results from the China Health and Retirement Longitudinal Study | 10.1002/art.39465 | 2016 | 0 | 30 |
| 6 | The global burden of hip and knee osteoarthritis: estimates from the global burden of disease 2010 study | 10.1136/annrheumdis-2013-204763 | 2014 | 0 | 28 |
| 7 | OARSI guidelines for the non-surgical management of knee, hip, and polyarticular osteoarthritis | 10.1016/j.joca.2019.06.011 | 2019 | 0.01 | 28 |
| 8 | Osteoarthritis: toward a comprehensive understanding of pathological mechanism | 10.1038/boneres.2016.44 | 2017 | 0.01 | 26 |
| 9 | Glucosamine, Chondroitin Sulfate, and the Two in Combination for Painful Knee Osteoarthritis | 10.1056/NEJMoa052771 | 2006 | 0 | 21 |
| 10 | 2019 American College of Rheumatology/Arthritis Foundation Guideline for the Management of Osteoarthritis of the Hand, Hip, and Knee | 10.1002/art.41142 | 2020 | 0 | 21 |
