# Supplementary material for: Research on Herbal Therapies for Osteoarthritis in 2004–2022: A Web of Science-Based Cross-Sectional Bibliometric Analysis
Source: Evid Based Complement Alternat Med. 2022 Jul 30;2022:6522690. doi: 10.1155/2022/6522690 (PMC9356781; doi:10.1155/2022/6522690)
Supplement: Supplementary Materials — Figure S1 Flowchart of literature search and selection. Figure S2 Trends of publications in the field of herbal therapies for OA from 2004 to 2022. Table S1 Top keywords (n ≥ 80) related to herbal therapies for OA. Table S2 Top 10 references related to herbal therapies for OA. Table S3 The clusters of cocited references in herbal therapies for OA. [file 6522690.f1.zip › Table S3 (1).docx]

TABLE S3 The clusters of co-cited references in herbal therapies for OA.

| **ClusterID** | **Size** | **Silhouette** | **Mean (Year)** | **Label** |
| --- | --- | --- | --- | --- |
| 0 | 166 | 0.861 | 2017 | network pharmacology |
| 1 | 117 | 0.927 | 2010 | chronic disorder |
| 2 | 107 | 0.879 | 2013 | mesenchymal stem cell |
| 3 | 97 | 0.951 | 2004 | clinical pharmacology |
| 4 | 71 | 0.999 | 2005 | herbal medicinal product |
| 5 | 66 | 0.968 | 2002 | biological basis |
| 6 | 58 | 0.942 | 2010 | chronic knee pain |
