# Supplementary figures and images for: Research on Herbal Therapies for Osteoarthritis in 2004–2022: A Web of Science-Based Cross-Sectional Bibliometric Analysis
Source: Evid Based Complement Alternat Med. 2022 Jul 30;2022:6522690. doi: 10.1155/2022/6522690 (PMC9356781; doi:10.1155/2022/6522690)

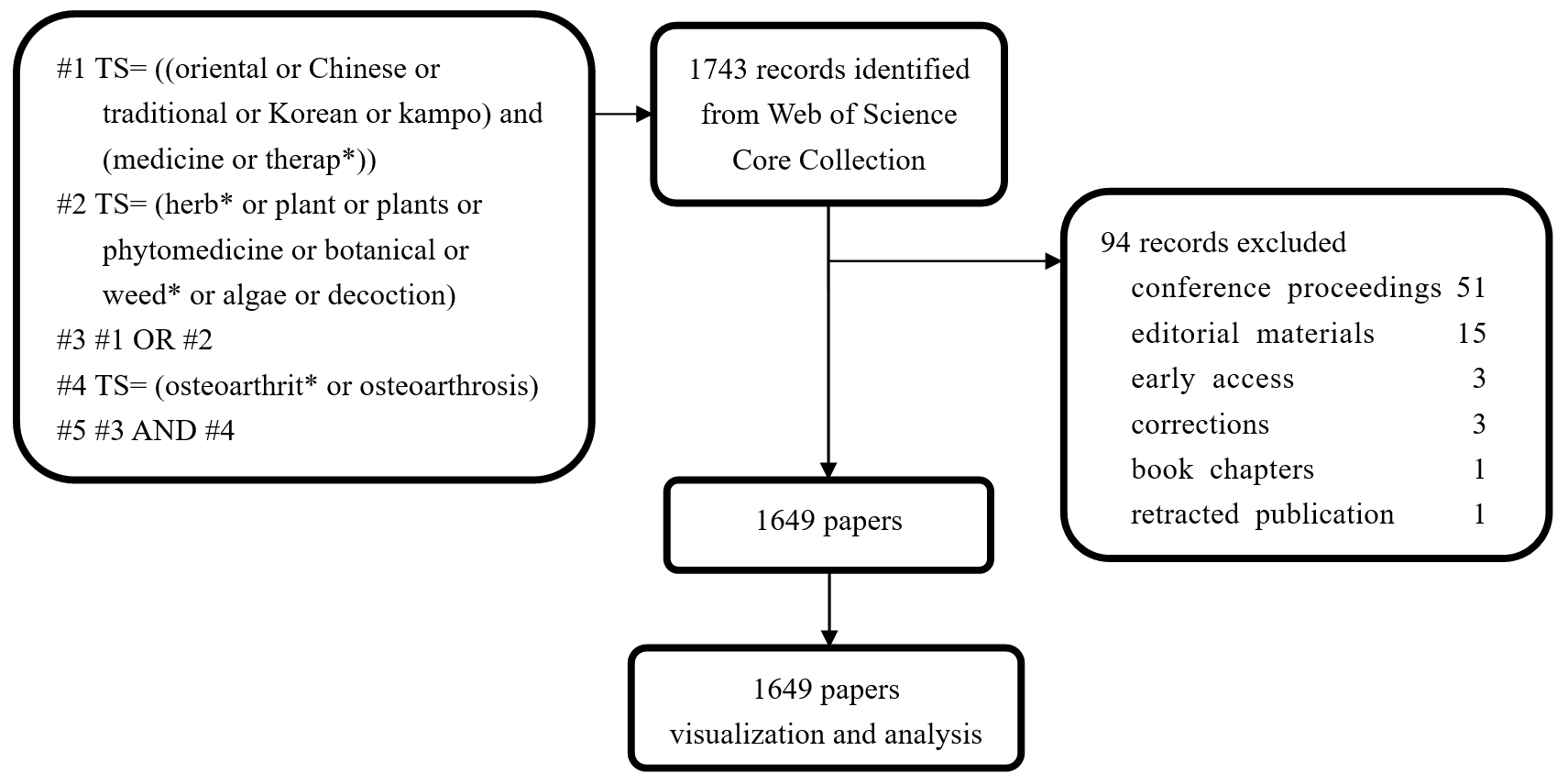


FIGURE S1 Flowchart of literature search and selection.

Supplement: Supplementary Materials — Figure S1 Flowchart of literature search and selection. Figure S2 Trends of publications in the field of herbal therapies for OA from 2004 to 2022. Table S1 Top keywords (n ≥ 80) related to herbal therapies for OA. Table S2 Top 10 references related to herbal therapies for OA. Table S3 The clusters of cocited references in herbal therapies for OA. [file 6522690.f1.zip › Figure S1 (1).docx]
